# Estimating invariant dimensions in V2

**DOI:** 10.1186/1471-2202-14-S1-P302

**Published:** 2013-07-08

**Authors:** Haruo Hosoya, Kota S Sasaki, Izumi Ohzawa

**Affiliations:** 1 Neural Information Analysis Laboratories, ATR International, Keihanna, Kyoto 619-0288, Japan; 2 Presto, Japan Science and Technology Agency, Chiyoda, Tokyo, Japan; 3 Graduate School of Frontier Biosciences, Osaka University, Toyonaka, Osaka 560-8531, Japan

## 

Our visual system can robustly detect an important feature in a scene, such as the identity of an object, even when its retinal image can vary substantially. Neural basis for such invariant recognition is thought to be the common property in visual cortex where a cell retains its activity even with large changes in stimuli along a certain dimension. For example, a face-selective cell may keep firing when the viewing angle of the face is changed; a V1 complex cell may remain active when the phase of an oriented grating is changed. A modern approach to identify such invariant dimensions is a semi-automatic analysis of responses to a large number of randomly chosen stimuli; a well-known method is spike-triggered covariance analysis (STC), which has successfully estimated receptive field elements with distinct phases for V1 complex cells [1].

This work aims at generalizing the previous semi-automatic method to reveal invariant dimensions in V2. STC is not suitable for direct applications to V2 cells since these typically have a much higher-degree of nonlinearity compared to V1 cells and since a large number of parameters are needed to characterize V2 cells and therefore STC requires an unrealistic amount of data. Instead, our approach is to assume a population model of V1 cells and analyze their outputs with respect to the responses of a V2 cell, where the latter part uses a Bayesian version of STC [2] for incorporating prior knowledge such as smoothness of receptive field shapes. Previous work on hierarchical analysis of V2 used only a first-order analysis on top of a V1 population model and therefore was not capable of revealing invariant dimensions in V2 [3,4].

We applied our method to a publicly available dataset of 127 V2 cells responding to a large number of natural images [3]. Our analyses revealed that V2 cells often had several invariant dimensions. To characterize these dimensions, we analyzed a quadratic model yielded by Bayesian STC to generate a set of abstract forms of sample stimuli producing optimal or near-optimal responses. The generated set exposed that the invariant dimensions typically represented positional translation, rotational transformation, expansion, compression, or their combination. Quantitative analysis showed a wide variety of invariant dimensions represented in V2, including large transformations like positional translations with the stimulus size, rotations with 45 degrees, and double size changes. Although invariant dimensions of similar kinds are known in higher visual areas, such dimensions have not been reported previously in V2, suggesting that complex invariant representations may already start as early as in V2.
